# The role of probiotics, prebiotics, and synbiotics in the treatment of inflammatory bowel diseases: an overview of recent clinical trials

**DOI:** 10.3389/fsysb.2025.1561047

**Published:** 2025-04-16

**Authors:** Fayez Yassine, Adam Najm, Melhem Bilen

**Affiliations:** ^1^ Department of Experimental Pathology, Immunology, and Microbiology, Faculty of Medicine, American University of Beirut, Beirut, Lebanon; ^2^ Faculty of Medicine, American University of Beirut Medical Center, Beirut, Lebanon

**Keywords:** probiotics, prebiotics, synbiotics, IBD, *Bifidobacterium*, *Lactobacillus*, fructooligosaccharides

## Abstract

**Background:**

The increasing incidence of inflammatory bowel diseases (IBD) over the last two decades has prompted the need to create new types of therapeutic interventions. The gut microbiome has emerged as a key component in the prognosis and pathophysiology of IBDs. The alteration or dysbiosis of the gut microbiome has been shown to exacerbate IBDs. The bacterial composition of the gut microbiome can be modulated through the usage of probiotics, prebiotics, and synbiotics. These interventions induce the growth of beneficial bacteria. Additionally, these interventions could be used to maintain gut homeostasis, reduce the inflammation seen in these morbidities, and strengthen the gut epithelial barrier.

**Methods:**

The literature review was conducted in October 2024 using PubMed, Scopus, and Google Scholar screening for recent clinical trials in addition to reviews relevant to the topic.

**Aims:**

This review aims to summarize the recent clinical trials of probiotics, prebiotics, and synbiotics in IBD patients highlighting their potential benefits in alleviating symptoms and enhancing the quality of life.

**Conclusion:**

Certain probiotic formulations such as single strain ones consisting of *Lactobacillus,* or mixed-strain combinations of *Lactobacillus* and *Bifidobacterium*, prebiotic compounds such as fructooligosaccharides, and synbiotic combinations of both have proven effective in improving the clinical, immunological, and symptomatic aspects of the disease course. While promising, these findings remain inconclusive due to inconsistent study designs, small sample sizes, and varying patient responses. This emphasizes the need for larger, well-controlled trials to determine their clinical efficacy.

## 1 Introduction

The gut microbiota is a complex and diverse community of microorganisms that live in the digestive tract of humans and animals (Gomaa, 2020). The human intestinal microbiome consists of over 1,000 species of bacteria and other microorganisms (Martyniak et al., 2021). The total number of commensal microorganisms is approximately equal to the number of human eukaryotic cells, with an estimated ratio of 1:1 (Sender et al., 2016). The commensal bacteria of the gut play a role in fermenting complex fibers and carbohydrates (Jandhyala et al., 2015), producing vitamins (LeBlanc et al., 2013), and providing protection against possible invading pathogens (Panwar et al., 2021). These commensal bacteria respond to host hormones and chemicals; in response, they produce metabolites that maintain homeostasis in the gut, influence immune response maturation and host energy metabolism, and preserve the mucosal integrity (Zheng et al., 2020; Nieuwdorp et al., 2014; Schreiber et al., 2024).

The gut microbiota is dominated mainly by 6 different phyla that include *Firmicutes, Bacteroidetes, Actinobacteria, Proteobacteria, Verrucomicrobia* and *Fusobacteria* (Ross et al., 2024; Hasan and Yang, 2019). In particular, the phylum *Proteobacteria* is characterized by the prevalence of opportunistic pathogens including *Escherichia coli*, *Salmonella*, *Campylobacter*, *Klebsiella*, and *Shigella* which have been implicated in both metabolic and inflammatory disorders (Rizzatti et al., 2017). Conversely, beneficial bacteria include those of the genera *Lactobacillus*, *Faecalibacterium*, and *Roseburia* belonging to the *Firmicutes* phylum (Lindstad et al., 2021; Linares et al., 2016; Nie et al., 2021; Martín et al., 2023). Additionally, the genus *Bifidobacterium*, classified under the *Actinobacteria* phylum, is also recognized as a beneficial member of the gut microbiota (Linares et al., 2016). These microbes play several essential roles including but not limited to the production of key metabolites such as butyrate (Valdes et al., 2018).

The intestinal microbiome is easily altered by internal or external factors such as diet, aging, or antibiotic usage (Ross et al., 2024; Hasan and Yang, 2019). A change in the normal composition of a healthy microbiome is known as dysbiosis (Weiss and Hennet, 2017). Most of these dysbiotic states are transient leading to temporary symptoms (Blumstein et al., 2014).

However, in a small portion of cases, these dysbiotic changes can be permanent and lead to the emergence of chronic diseases or symptoms (Blumstein et al., 2014). The illnesses that can arise from a prolonged or permanent state of dysbiosis include gastrointestinal illnesses such as colorectal cancer (Rebersek, 2021) and inflammatory bowel diseases (IBD) (Yu, 2018). Dysbiosis may also exacerbate previous existing intestinal or extra-intestinal diseases such as cardiovascular diseases (Nesci et al., 2023).

Dysbiosis can be reversed or modulated through a plethora of interventions that include antibiotics, probiotics, prebiotics, postbiotics, and even fecal microbiota transplantation (Fong et al., 2020). The most notable of these interventions are probiotics which have gained traction over the past decades (Kim et al., 2019). According to the expert panel from the International Scientific Association for Probiotics and Prebiotics (ISAPP), probiotics are defined as “live microorganisms which when administered in adequate amounts confer a health benefit on the host” (Hill et al., 2014). There is an abundance of literature illustrating the role of probiotics in the treatment of numerous disorders such as ulcerative colitis (UC), Crohn’s disease (CD), diarrhea, irritable bowel syndrome (IBS), obesity, cancer, and insulin resistance (Petrariu et al., 2024; Cerdó et al., 2019; Kijmanawat et al., 2019; Whelan and Quigley, 2013).

**FIGURE 1:**
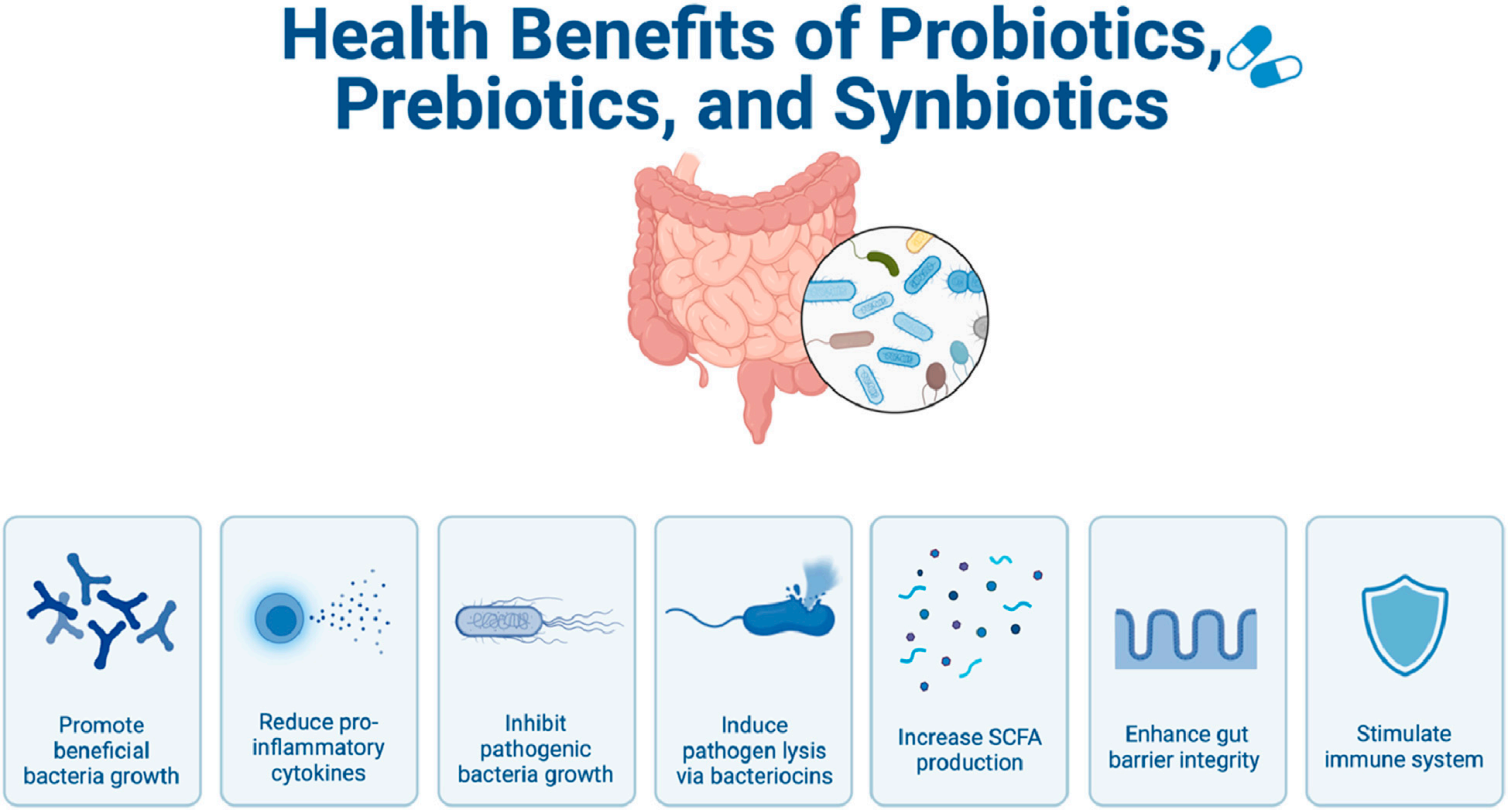
An illustration demonstrating the health benefits of probiotics, prebiotics, and synbiotics on the gut microbiome. This illustration was created using BioRender.

Prebiotics are a substrate that is selectively utilized by the beneficial host organisms to confer a health benefit (Yadav et al., 2022). The combination between a probiotic and prebiotic is referred to as a synbiotic (Yadav et al., 2022). Synbiotics, like probiotics, have been shown to restore the normal gut flora and treat inflammation along with a wide array of morbidities (Singh et al., 2023; Jadhav et al., 2023). In certain instances, synbiotics have been shown to have a greater efficacy than either probiotics or prebiotics used in isolation (Mohanty et al., 2018). Figure 1 represents the health benefits of probiotics, prebiotics, and synbiotics on the gut microbiome.

This narrative review aims to provide an overview on probiotics, prebiotics, synbiotics and their potential to treat IBDs. It will summarize clinical data and trials on probiotics, prebiotics, and synbiotics on IBD patients.

## 2 Methods

The literature review was conducted in October 2024, providing an overview on probiotics, prebiotics, synbiotics as well as relevant clinical trials. The utilized databases include PubMed, Scopus, and Google Scholar. The results were retrieved using the following key words: “Probiotics,” “Prebiotics,” “Synbiotics,” “Inflammatory bowel disease,” “VSL#3,” “Bifidobacterium,” “*Lactobacillus,*” as well as their relevant MeSH terms.

This strategy aimed to explore articles that used probiotics, prebiotics, or synbiotics as an intervention or adjunct therapy for the treatment of IBDs while providing an overview for the relevant mechanisms. Articles were selected based on their relevance and findings pertinent to this review. The inclusion criteria encompassed studies without restrictions on their type or country of origin, provided they were written in or translated into English.

In addition to primary research articles, relevant reviews were added to provide a comprehensive overview of the topic. References from selected articles were also examined to identify significant studies. This narrative review involved a thorough examination of each article’s relevance and validity to ensure a rigid and extensive synthesis regarding probiotics, prebiotics, synbiotics, and their potential role in treatments.

## 3 The immune and microbial factors in inflammatory bowel diseases

IBDs are of two types: CD and UC (Qiu et al., 2022). Over the past few decades, the incidence of these morbidities in Europe and North America has remained relatively constant, while their occurrence in newly industrialized countries has continued to increase rapidly (Roda et al., 2020; Kaplan, 2015; Ng et al., 2017). Despite the etiology of these diseases remaining largely unknown, a variety of factors are thought to be involved including genetic susceptibility, immune factors, and the gut microbiota (Qiu et al., 2022).

The inflammatory nature of both CD and UC is caused by an overly aggressive immune response to a subset of commensal enteric gut microbes (Sartor, 2006). On one hand, the immune response in CD is thought to be driven by Type-1 helper cell response (Zhang and Li, 2014). On the other hand, Type 2 helper cells are thought to instigate the immune response in UC (Zhang and Li, 2014). In terms of microbial composition, both CD and UC patients experience a decrease in microbial abundance, diversity, and stability (Carding et al., 2015). Specifically, there is a reduction in *Firmicutes* and *Bacteroidetes* accompanied by an increase in *Proteobacteria* and *Actinobacteria* (Alshehri et al., 2021). Additional studies also noted a decrease in anaerobic bacteria such as *Bifidobacterium* and *Lactobacillus* alongside an increase in *Escherichia* and *Enterococci* (Martyniak et al., 2021). These dysbiotic alterations can lead to a weakened intestinal epithelial barrier resulting in increased luminal antigen uptake and continuous immune activation (Alshehri et al., 2021).

In CD, the entire gastrointestinal tract can be affected with the most common segments affected being the terminal ileum and the colon (Torres et al., 2017). Inflammation in CD is typically segmental, asymmetrical, and transmural (Torres et al., 2017). This differs from UC, where inflammation is typically restricted to the mucosal surface (Ordás et al., 2012). Additionally, the primary organ affected by UC is the colon (Meier and Sturm, 2011). Treatments for IBDs typically include aminosalicylates, corticosteroids, immunomodulators, biologics, and probiotics (Cai et al., 2021). In addition to these conventional therapies, a recent advancement in the treatment of IBD involves the application of bio-nanomaterials which enable targeted drug delivery, control the release of the pharmacological compounds, and provide stimuli-responsive therapy while reducing side effects (Stojanov and Berlec, 2024).

## 4 Role of probiotics, prebiotics, and synbiotics in gut health

### 4.1 Role of probiotics

Historically, the consumption of probiotics began when early civilization humans began to consume fermented foods (Yadav et al., 2022). The term probiotics was coined to reflect the work of Elie Metchnikoff (Anukam and Reid, 2007). Metchnikoff made the ground-breaking observation that the regular consumption of lactic acid bacteria in fermented dairy products such as yogurt was associated with enhanced health and longevity in the Bulgarian Peasant populations (Anukam and Reid, 2007).

The latest definition of probiotics considers them to contain living organisms that must be ingested in a sufficient amount to have a positive effect on health that is not limited to the nutritional effects (Kechagia et al., 2013). The first available probiotics contained only one species of microorganisms, mainly those belonging to the *Saccharomyces* or *Lactobacillus* genera (Wieërs et al., 2020).

Numerous studies have shown that probiotics can modulate the microbial flora composition of the gut, inhibit pathogenic bacteria from colonizing the gut, and assist the host in building a healthy intestinal mucosa (Chandrasekaran et al., 2024). There are many mechanisms by which probiotics exert their beneficial effects (Chandrasekaran et al., 2024). Probiotics have been shown to increase the number of beneficial bacteria in the intestine by promoting the growth of endogenous desirable microbial populations as well as their own growth (Fassarella et al., 2021). Another mechanism by which probiotics favourably affect the gut is through competitive exclusion (Monteagudo-Mera et al., 2019). This process promotes the growth of beneficial bacteria and inhibits the growth of pathogenic ones (Monteagudo-Mera et al., 2019).

Additionally, probiotics have been shown to enhance or restore the gut barrier function (Abraham and Quigley, 2017). This happens by inhibiting the apoptosis of intestinal epithelial cells and promoting the synthesis of proteins that are critical components of tight junctions (Yan and Polk, 2002; Rose et al., 2021). Probiotics have also been shown to exhibit anti-inflammatory effects modulating local as well as mucosal inflammation (Cristofori et al., 2021). Studies have unveiled the ability of probiotics to reduce inflammation by increasing the production of certain anti-inflammatory cytokines such as interleukin 10 (IL-10) and transforming growth factor-beta (TGF-ß) while reducing the expression of pro-inflammatory cytokines such as interferon gamma (IFN-γ) and IL-1 (Cristofori et al., 2021). A potential mechanism for the modulation of inflammatory markers may involve the inhibition of the nuclear factor-kappa B (NF-κB) pathway (Mahapatro et al., 2023).

Probiotics have been shown to induce the expression of Immunoglobulin A (IgA) and stimulate the maturation of the humoral immune system (Maldonado Galdeano et al., 2019). They can stimulate the macrophages and dendritic cells which are immune cells that can aid in the identification and elimination of pathogens (Shi et al., 2017).

Another way through which probiotics exert their beneficial effects is through the creation of an acidic milieu that is inimical to proinflammatory bacteria but supportive to the growth of beneficial species of bacteria such as *Lactobacilli* and *Bifidobacteria* (Abraham and Quigley, 2017). Their ability to colonize the gastrointestinal tract is further aided by their production of bacteriocins which are molecules that inhibit the growth of pathogenic bacteria (Gillor et al., 2008).

### 4.2 Role of prebiotics

Based on a December 2016 microbiology expert panel, the term “prebiotics” refers to molecules that can be manipulated by the host microbiota to achieve health benefits like preventing disease or improving outcomes (Gibson et al., 2017). While more inclusive than the initial description of prebiotics which had narrowed them down to non-digestible oligosaccharides, even initial views stated that these molecules must preferentially enhance the development of certain beneficial gut bacteria over their non-beneficial counterparts (Gibson and Roberfroid, 1995). As opposed to the live organisms forming probiotics, prebiotics consist of substances that can supplement bacterial growth most commonly stemming from natural sources with plant-based oligosaccharides being the most common (Oniszczuk et al., 2021). Several everyday foods have been shown to exhibit prebiotic properties such as oats, soybeans, and honey (Olas, 2020) given their biochemical composition, but it must be noted that these are only some of the vast possible sources of the prebiotic compounds to be mentioned. Several forms of carbohydrates have demonstrated prebiotic potential such as lactulose, galactooligosaccharides, fructooligosaccharides, maltooligosaccharides, cyclodextrins, and lactosaccharose as well as fructans such as inulin and oligofructose (Markowiak and Śliżewska, 2017). In most cases, the main bacterial agents of the attempts to modify the gut microbiome are of the *Bifidobacterium* and *Lactobacillus* strains which metabolize the carbohydrate-based prebiotics to short-chained fatty acids (SCFAs) which decreases the gut pH (Gibson and Wang, 1994). This decrease in pH which the aforementioned bacteria tolerate helps inhibit the proliferation of pathogenic strains. Another relevant example is the water extract from silver fir (*Abies alba*) wood which represents a source of lignans and polyphenols. Its rich carbohydrate content explains its potential to act as a prebiotic for multiple strains of *Lactobacillus* (Stojanov et al., 2021).

Dietary habits have been shown to modulate the gut microbiome composition which can be divided into enterotypes. In a study by Wu et al., fecal analysis after controlled feeding of 10 subjects revealed two main enterotypes based on the type of dietary intake (Wu et al., 2011). Changes occurred within 24 h of the diet before reverting to a composition resembling the baseline. High fat/low fiber diets led to the prevalence of the *Bacteroides*-dominant enterotype, while the low fat/high fiber diets instead promoted a *Prevotella*-dominant enterotype (Wu et al., 2011). This proves that dietary fibers, many of which are considered prebiotics based on their promotion of certain bacterial strains’ growth, could modulate gut microbiome composition.

Prebiotics can reach the lower GI tract intact due to their resistance to digestion by gastric acid or mammalian hydrolases and to absorption in the upper GI tract (Ashaolu et al., 2021). Once in the colon, their selective fermentation by beneficial bacteria will promote those strains’ growth in favor of a more balanced microbiota composition (Ashaolu et al., 2021). Furthermore, a direct result of microbiome metabolism of prebiotics is the production of SCFAs which have been shown to regulate gut microbiome homeostasis and contribute to the pathogenesis of multiple disease and inflammatory processes when deficient (Fusco et al., 2023). SCFAs produced in the gut include acetate, propionate, and butyrate which are the most abundant anions in the human body (Portincasa et al., 2022).

Patients with UC have been shown to have a lower prevalence of butyrate-producing bacteria in their gut such as *Roseburia hominis* and *Faecalibacterium prausnitzii* with their abundance being inversely related to disease activity (Machiels et al., 2014). The SCFAs produced by species selected-for by prebiotics can relieve the chronic inflammation seen in multiple inflammatory disorders (Liu et al., 2023). Butyrate and propionate have been shown to minimize the recruitment of monocytes and neutrophils through the inhibition of the inducible expression of adhesion molecules and chemokine production as part of an anti-inflammatory mechanism that could benefit patients with autoimmune diseases (Vinolo et al., 2011). Butyrate in particular suppresses LPS- and cytokine-mediated production of pro-inflammatory mediators such as TNF-α, IL-6, and nitric oxide (NO) while upregulating the release of anti-inflammatory cytokines such as IL-10 (Vinolo et al., 2011). In addition, the SCFAs synthesized through prebiotic administration have been shown to decrease gut pH due to protons being a byproduct of the reaction (den Besten et al., 2013). This pH change could be gut-protective given that some beneficial bacteria are selected for under weakly acidic pH, and most pathogenic bacteria would be selected against in this acidic environment as they thrive in more pH-neutral conditions (Yamamura et al., 2023). Butyric acid-producing bacteria like *Faecalibacterium* and *Roseburia* have been shown to grow better in weakly acidic environments rather than at neutral pH (Walker et al., 2005).

Prebiotics also help preserve intestinal barrier integrity through upregulating tight junction proteins involved in the assembly of the zonula occludens ZO-1 (Wongkrasant et al., 2020). This was proven to be through the AMP-activated protein kinase promotion of tight junction assembly through a calcium sensing receptor (CaSR)-phospholipase C (PLC)- Ca^2+^/calmodulin-dependent protein kinase kinase-β (CaMKKβ) pathway in an intestinal epithelial cell model (Wongkrasant et al., 2020).

### 4.3 Role of synbiotics

**FIGURE 2:**
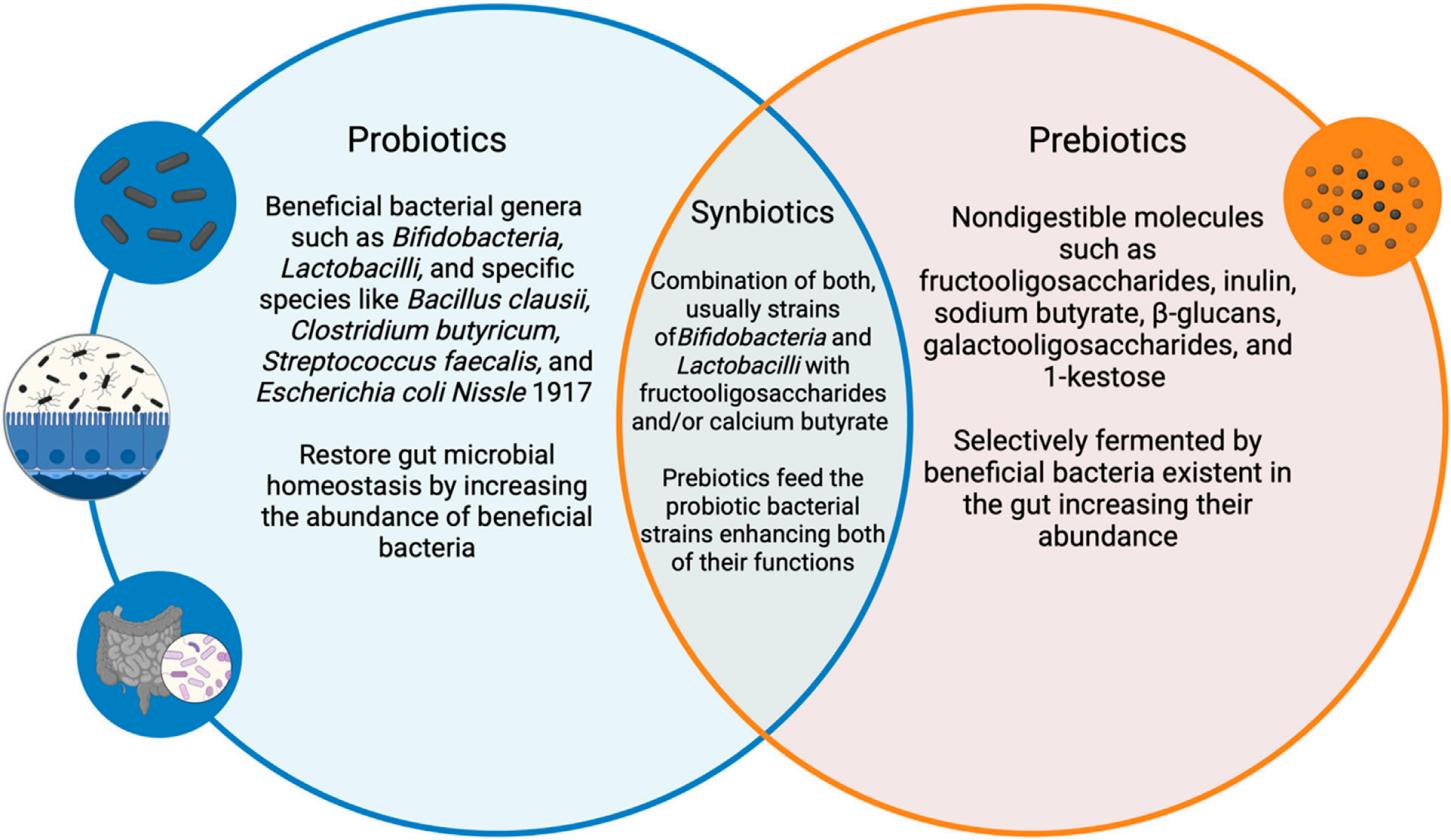
An illustration demonstrating the relationship between probiotics, prebiotics and synbiotics along with relevant examples. This illustration was created using BioRender.

The most recent definition of “Synbiotics” by the International Scientific Association for Probiotics and Prebiotics (ISAPP) described them as “a mixture comprising live microorganisms and substrate(s) selectively utilized by host microorganisms that confers a health benefit on the host” where the host microbes can either be the resident microflora of the host ingesting the synbiotic or the exogenous “host” microbes within the synbiotic mixture (Wu et al., 2011). This definition combines both concepts of probiotics as the live microbes and prebiotics as the substrates selectively promoting those microbes as well as the resident flora into one holistic treatment (Swanson et al., 2020). This implies that consuming a combination of both confers an increased microbial survival of the probiotic formulation as well as a better chance for the established healthy microflora to thrive (Takahashi et al., 2018). Figure 2 highlights the relationship between probiotics and prebiotics and how they combine to form synbiotics.

## 5 Intervention of probiotics, prebiotics, and synbiotics in IBD: evidence from clinical trials and observational studies

### 5.1 Intervention of probiotics

Numerous clinical trials and observational studies have assessed the effectiveness of probiotics in inducing and maintaining remission in both UC and CD patients. The selected studies were grouped into different categories based on similar outcome measures.

#### 5.1.1 Clinical activity and endoscopic improvement in UC

Several of the selected studies demonstrated significant improvements in clinical indices and endoscopic outcomes in UC patients. A study conducted by Agraib et al. tested the efficacy of a probiotic blend containing nine *Lactobacillus* and five *Bifidobacterium* species on UC patients for 6 weeks (Agraib et al., 2022). The study reported a significant increase in the partial Mayo score, stool frequency, and global assessment (Agraib et al., 2022). Another randomized double blind clinical trial tested the efficacy of *Escherichia coli Nissle* 1917 and mesalazine against mesalazine and placebo only (Park et al., 2022). The patients that were treated with the probiotic and mesalazine observed a significant decrease in their partial Mayo score and improved abdominal pain score as well as higher rates of endoscopic remission compared to those receiving mesalazine with the placebo (Park et al., 2022). However, this study also reported no significant difference in clinical remission rates, and stool frequency (Park et al., 2022). An additional randomized open label clinical trial tested the efficacy of *Lactobacillus rhamnosus GG* as a regular dose or double dose (Pagnini et al., 2023). The study observed significant improvement in the clinical activity and endoscopic scores in the UC patients treated with the probiotic over 4 weeks irrespective of the dosage administered (Pagnini et al., 2023). Conversely, a study testing the efficacy of a probiotic mixture containing eight strains (four *Lactobacillus*, three *Bifidobacterium*, and one *Streptococcus)* did not observe significant changes in the clinical indices (Tamizifar et al., 2023). After 16 weeks of treatment, the calprotectin levels, Mayo, and Lichtiger scores did not significantly change demonstrating variability in clinical outcomes (Tamizifar et al., 2023). Additionally, another study reported no significant improvement in the CD activity index, IBS severity, gastrointestinal symptoms, and fecal calprotectin despite probiotic administration for 4 weeks (Tomita et al., 2023). A different study reported an improved Simple Clinical Colitis Activity Index (SCCAI) but no significant difference in the Crohn’s disease activity index (CDAI) score between the 2 cohorts (Bamola et al., 2022).

#### 5.1.2 Modulation of inflammatory markers and gut microbiota

Five of the selected studies focused on the biochemical and microbiological effects of probiotic therapy. Agraib et al. noted that despite the pro-inflammatory cytokines IL-1, IL-6, tumor necrosis factor- α (TNF- α) remaining unchanged, there was a significant increase in the anti-inflammatory cytokine IL-10 in the probiotic group compared to the placebo (Agraib et al., 2022). Another double blind randomized controlled trial (RCT) study studied the effects of *Bacillus clausii* UBBC-07 in both UC and CD patients for 4 weeks (Bamola et al., 2022). The study also reported an increased abundance of the *Firmicutes, Lactobacillus, Bifidobacterium*, and *Faecalibacterium* in the gut microbiota composition (Bamola et al., 2022). Additionally, the study reported a significant increase in anti-inflammatory cytokines, alongside a marked decrease in pro-inflammatory cytokines (Bamola et al., 2022). A third study conducted by Shen et al. demonstrated the ability of the probiotic to regulate the intestinal microbiota (Shen et al., 2024). In particular, there was significant increase in the abundance of *Lactobacillus acidophilus* and *Bifidobacterium longum* along with a decrease of *Bacteroides vulgatus* (Shen et al., 2024). This study also reported a significant reduction in inflammation alongside an improvement in immune and intestinal barrier function (Shen et al., 2024). Conversely, another study demonstrated a lack of gut microbial alterations despite probiotic administration in the CD population (Tomita et al., 2023). A fifth study also demonstrated the lack of gut microbiome alteration despite probiotic intervention (Park et al., 2022).

#### 5.1.3 Quality of life and symptom relief

Lee et al. conducted an observational study composed of 43 UC patients in endoscopic remission (Lee et al., 2022). The patients were given a probiotic blend consisting of *Lactobacillus acidophilus*, *Clostridium butyricum*, *Bacillus mesentericus*, and *Streptococcus faecalis* (Lee et al., 2022). The intervention was shown to significantly improve the stool frequency, Bristol scale, and short inflammatory bowel disease questionnaire scores in UC patients (Lee et al., 2022). However, there was no alteration in the abdominal pain (Lee et al., 2022). Another double blind RCT studied the effects of a probiotic mixture consisting of nine *Lactobacillus* and five *Bifidobacterium* strains in the UC population (Rayyan et al., 2023). The study demonstrated that over 6 weeks, the UC patients receiving the probiotic exhibited significant enhancement in the systemic, social, bowel, and emotional domains in addition to a significant improvement in the total SIBDQ score (Rayyan et al., 2023). A study conducted on the CD population reported significant improvements in the disease specific quality of life, mental summary, and Hospital Anxiety and Depression Scale (HADS) score (Tomita et al., 2023). Another study conducted by Bamola et al. also reported a significant improvement in the psychological parameters in the probiotic group (Bamola et al., 2022). However, one study reported no differences in the inflammatory bowel disease questionnaire scores (IBDQ) between the two groups (Park et al., 2022).

Overall, the use of probiotics in IBD shows promise, but results are mixed. Several studies reported improvements in clinical activity, endoscopic outcomes, inflammatory markets, and gut microbiota composition, especially in UC patients. However, other trials found no significant changes in these indices and criteria highlighting the variability in patient response and in the efficacy of different probiotic formulations.

The probiotic studies in IBD are presented in Table 1.

**TABLE 1:** Studies evaluating probiotics in IBD patients.

Study Reference	Study design	Subject	Intervention	Number of patients	Duration of the study	Dosage	Outcome
Agraib et al. (2022)	RCT-double blind	Mild to moderate UC	Nine *Lactobacillus* and 5 *Bifidobacterium* species vs. Placebo	30	6 weeks	1 × 10^10^ CFU/g; daily	Significant induction of remission in the subject group compared to the control, significant improvement in partial Mayo score, stool frequency and global assessment; significant increase in IL-10 in the probiotic group No significant changes in the levels of IgG, IgM, IgA, IL-1, IL-6, and TNF-α between the 2 cohorts
Bamola et al. (2022)	RCT-double blind	UC and CD patients	*Bacillus clausii* UBBC-07 vs. Placebo	110	4 weeks	2 billion per capsule; twice a day	Significant increase in *Firmicutes*, *Lactobacillus*, *Bifidobacterium*, and *Faecalibacterium* in the probiotic group; Significant increase in anti-inflammatory cytokines and decrease in pro-inflammatory ones and significant improvement in psychological parameters in the probiotic group. Significant improvement in the SSCAI but not the CDAI score
Lee et al. (2022)	Observational study	UC patients in endoscopic remission	*Lactobacillus acidophilus*, *Clostridium butyricum*, *Bacillus mesentericus*, and *Streptococcus faecalis*	43	4 weeks	*Lactobacillus acidophilus*:75 mg, *Clostridium butyricum*:25 mg, *Bacillus mesentericus*:25 mg, *Streptococcus faecalis*:5 mg per capsule; 3 capsules a day	Significant improvement in stool frequency, Bristol scale, SIBDQ scores and quality of life; no significant changes in AP.
Park et al. (2022)	RCT-double blind	UC	*Escherichia coli Nissle* 1917 and mesalazine vs. mesalazine and placebo	134	8 weeks	2.5 × 10^9^ CFU per capsule; 1 capsule per day from day to day 4, then two capsules per day from day 5 onwards	Significant decrease in Mayo scores and improvement in AP scores in the probiotic group at weeks 4 and 8, with more patients achieving endoscopic remission. No significant differences in clinical remission, SF, or diversity measures. No difference in IBDQ scores, but significant prevention of QoL decline in the probiotic group
Pagnini et al. (2023)	RCT-open label	Mild to moderate UC	*Lactobacillus rhamnosus GG* (ATCC 53103) as regular dose or double dose	76	4 weeks	2 doses:1.2 or 2.4 × 10^10^ CFU taken once a day	Significant improvement in clinical activity and endoscopic score improvement after LGG treatment; no significant difference in the clinical outcomes between the different doses
Rayyan et al. (2023)	RCT-double blind	UC	Nine *Lactobacillus* and five *Bifidobacterium* species vs. Placebo	24	6 weeks	2.5 × 10^9^ CFU/g; single dose a day	Significant improvement in the systemic, social, bowel, and emotional domains as well as total SIBDQ in the probiotic group
Tamizifar et al. (2023)	RCT-double blind	Mild to moderate UC	4 *Lactobacillus*, 3 *Bifidobacterium* and 1 *Streptococcus* species vs. Placebo	60	16 weeks	4.5 × 10^11^ CFU for each capsule; two capsules per day	No significant differences were observed in the calprotectin, Mayo, and Lichtiger values between the probiotic and placebo groups
Tomita et al. (2023)	Prospective clinical trial	quiescent CD	*Bifidobacterium bifidum* G9-1	12	4 weeks	*Bifidobacterium bifidum* G9-1:24 mg per capsule administered 3 times per day	No significant improvement in CD activity index, IBS severity, gastrointestinal symptoms, physical summary, fecal calprotectin, or microbiome diversity; significant improvements in disease specific QOL, mental summary, and HADS score
Shen et al. (2024)	RCT	CD	*Bifidobacterium, Lactobacillus, Enterococcus* species with Mesalamine vs. Mesalamine only	96	4 weeks	1.0 × 10^6^ CFU/g; 3 capsules per day	Significant clinical efficacy, reduction in inflammation, increase in abundance of *Lactobacillus acidophilus* and *Bifidobacterium longum and decrease of Bacteroides vulgatus*, improvement in nutritional indicators, enhancement of immune function, and intestinal mucosal barrier function

Abbreviations: RCT, randomized controlled trial; CFU, colony forming unit; AP, abdominal pain; SF, stool frequency; QoL, quality of life.

### 5.2 Intervention of prebiotics

#### 5.2.1 Clinical remission and disease activity

An RCT conducted by Valcheva et al. assessed the effectiveness of administering different dosages of oligofructose-enriched inulin on 25 patients with mild-to-moderately active UC for 9 weeks (Valcheva et al., 2019). The high-dose group reported significantly higher clinical response and remission rates compared to the low-dose group. Pietrzak et al. conducted a double-blind RCT comparing the efficacy of sodium butyrate compared to a placebo to treat 72 pediatric patients with either UC or CD over 12 weeks (Pietrzak et al., 2022). The study revealed that most patients achieved clinical remission regardless of receiving the intervention or not. Both groups showed no significant differences in terms of their remission rates and median disease activities. Another study by Ikegami et al. studied the use of 1-kestose over 8 weeks compared to a placebo in a double-blind RCT on 40 mild-to-moderately active UC patients (Ikegami et al., 2023). The treatment group exhibited a significantly lower clinical activity index along with a significant improvement in clinical remission and response rates. However, the Ulcerative Colitis Endoscopic Index of Severity (UCEIS) scores were not significantly different amongst the treatment and control groups.

#### 5.2.2 Symptom relief and quality of life

In an RCT conducted by Nyman et al., the researchers compared the use of oat bran rich in β-glucans to a low-fiber alternative in treating 94 patients with UC in remission (Nyman et al., 2020). Over 24 weeks, the oat bran group achieved subjective health maintenance along with the prevention of symptomatic deterioration more effectively than the low-fiber group. While the relapse rates were similar in both groups, the control group did exhibit significantly more obstipation, reflux, and symptom burden. A different study by Facchin et al. addressed the quality-of-life changes of 49 patients with either UC or CD within 6 months of their diagnosis through an RCT that compared microencapsulated sodium butyrate treatments to a placebo (Facchin et al., 2020). After the 60-day period of the trial, the UC patients receiving the treatment experienced improvements in quality of life based on changes in their IBD Questionnaire (IBDQ) answers.

A paper published by Baghizadeh et al. presented an RCT that investigated the use of *Plantago major* (*P. major*) seeds compared to a roasted wheat flour control group to treat 61 patients with mild, moderate, and severe UC over 8 weeks (Baghizadeh et al., 2021). The *P. major* seeds were selected for their richness in prebiotic compounds such as tannins, coumarins, flavonoids, polyphenols, and gluten. The treatment group experienced significantly less severe abdominal tenderness, gastric pain, and gastroesophageal reflux compared to the control. While they did also exhibit a significant decrease of hematochezia, abdominal distention, and rectal pain compared to their own baseline, that difference was not significant compared to the changes in those values in the control group. Finally, in a non-placebo-controlled open label trial by Wilson et al., 17 patients with mildly active UC were given a galactooligosaccharide supplement daily over 6 weeks (Wilson et al., 2021). The patients experienced more normalized stools but no significant effects on clinical scores and inflammation. Their stools showed more normalized Bristol Stool Form Scale (BSFS) values along with the reduced incidence and severity of loose stools. However, they showed no significant changes in fecal calprotectin, SCCAI scores, and stool pH.

#### 5.2.3 Microbiota composition and SCFA production

Multiple of the selected studies investigated the effects of the interventions in affecting gut microbiota composition and changes in metabolism including SCFA production as an important indicator. In the trial using oligofructose-enriched inulin (Valcheva et al., 2019), the high-dose group experienced more colonic butyrate production which could be explained by the increase in abundance of *Bifidobacteriaceae* and *Lachnospiraceae*, yet this change was found to be unrelated to the decreased colitis. The researchers identified that microbiome metabolism changes such as butyrate production are more important than the microbiota composition itself. With regards to the oat bran supplement trial (Nyman et al., 2020), the treatment group had significantly higher fecal butyrate levels and lower serum low-density lipoprotein (LDL) compared to the control group who did not experience significant changes.

In the microencapsulated sodium butyrate study by Facchin et al. (2020), the UC group receiving treatment had more SCFA-producing strains in their stools such as *Lachnospiraceae* compared to their control. Moreover, the CD group receiving treatment exhibited an increase in stool *Butyricicoccus* compared to their respective control group. In terms of the galactooligosaccharide trial by Wilson et al. (2021), the treatment group’s stools showed no significant changes in stool SCFA levels even though patients with SCCAI ≤ 2 had an increased relative abundance of *Bifidobacterium* and *Christensenellaceae*. Finally, in the Ikegami et al. (2023) 1-kestose trial, SCFA values were unaffected regardless of the significantly reduced abundance of *Ruminococcus gnavus*.

The prebiotic studies in IBD are presented in Table 2.

**TABLE 2:** Studies evaluating prebiotics in IBD patients.

Study Reference	Study design	Subject	Intervention	Number of patients	Duration of the study	Dosage	Outcome
Valcheva et al. (2019)	RCT – not placebo-controlled, groups received different dosages of the same prebiotics	Mild-to-moderately active UC	Oligofructose-enriched inulin	25	9 weeks	7.5 g/day for the low-dose group and 15 g/day for the high-dose group	-Significantly higher clinical response and remission rates in the high-dose group - Increased colonic butyrate production in the high-dose group -Increased *Bifidobacteriaceae* and *Lachnospiraceae* abundance with the high dose yet unrelated to the decreased colitis -Microbiome metabolism changes understood to be more important than the composition variability
Nyman et al. (2020)	RCT – no true placebo but comparing the oat bran to a low-fiber wheat alternative	UC in remission	Oat bran (rich in β-glucans)	94	24 weeks	60 g per day of oat bran (equivalent to 12 g of dietary fiber or 6 g of β-glucans) for the active group and 5 g of dietary fiber (equivalent to <0.5 g of β-glucans) for the control group	-Significantly higher fecal butyrate and lower serum LDL in the oat bran group compared to no significant effect by the control diet. -Maintenance of subjective health and prevention of symptomatic deterioration in the oat bran group -Significantly increased obstipation, reflux, and symptom burden in the control group -Similar relapse rates in both groups
Facchin et al. (2020)	RCT – placebo-controlled, double-blind	UC and CD patients within 6 months of diagnosis	Microencapsulated sodium butyrate	49	60 days	3 capsules of sodium butyrate per day (1800 mg per day) during the main meals	- Increased SCFA producer strains such as *Lachnospiraceae* in the UC group with the intervention - Increased *Butyricicoccus* in the CD group with the intervention - Increased QoL in the UC group
Baghizadeh et al. (2021)	RCT – no true placebo but rather comparing the roasted *P. major* seeds to a roasted wheat flour control group	Mild, moderate, and severe UC patients	Roasted Plantago major seed rich in tannins, coumarins, flavonoids, polyphenols, and gluten	61	8 weeks	3,600 mg per day	- Significantly less severe abdominal tenderness, gastric pain, and gastroesophageal reflux in the *P. major* group compared to the control - Significantly less hematochezia, abdominal distention, and rectal pain compared to baseline but no significant difference to the control
Wilson et al. (2021)	Open-label study, non-placebo-controlled	Mildly active UC	Galactooligosaccharides	17	6 weeks	2.8 g per day	- Unchanged fecal calprotectin, SCCAI, SCFA, and stool pH - More normal BSFS proportions and reduced incidence and severity of loose stools along with frequency - Increased relative abundance of *Bifidobacterium* and *Christensenellaceae* but mostly in patients with an SCCAI ≤ 2 - Normalized stools but no significant effect on clinical scores and inflammation
Pietrzak et al. (2022)	RCT – prospective, placebo-controlled, multi-centre, double-blinded	Pediatric active UC or CD	Sodium butyrate	72	12 weeks	150 mg every 12 h	- Majority of patients achieved remission regardless of intervention - No significant differences in remission rates or median disease activity
Ikegami et al. (2023)	RCT – placebo-controlled, double-blind	Mild-to-moderate UC	1-kestose	40	8 weeks	5 g per dose twice daily	- Significantly lower clinical activity index as well as higher clinical remission and response rates in the treatment group - UCEIS not significantly different - Significantly reduced abundance of some bacteria such as *Ruminococcus gnavus* - SCFA insignificantly different amongst the groups

### 5.3 Intervention of synbiotics

#### 5.3.1 Clinical activity measures and laboratory markers

In a placebo-controlled RCT conducted by Kamarlı Altun et al. (2019), researchers studied the effects of combining six bacterial strains (two *Lactobacillus*, two *Bifidobacterium*, one *Enterococcus*, and one *Streptococcus*) along with fructooligosaccharides against a placebo to treat 40 patients with mild-to-moderately active UC. After the 8-week duration of the trial, the treatment group showed a significant decrease in serum C-reactive protein (CRP) and sedimentation values compared to baseline indicating a decrease in systemic inflammation. Moreover, they experienced significant improvement in their clinical parameters compared to the placebo group. While they showed significant improvement in clinical inflammatory markers and endoscopic disease activity compared to baseline, this was not significantly different to that of the placebo group.

Another study by Amiriani et al. (2020) investigated the use of seven bacterial strains (four *Lactobacillus*, two *Bifidobacterium*, and one *Streptococcus*) with fructooligosaccharides in a placebo-controlled, double-blinded RCT to treat 60 patients with mild-to-moderately active UC over 8 weeks. The group receiving the treatment labelled as Lactocare showed a significant reduction in their mean SCCAI compared to an insignificant reduction in the control group. While the percentage of patients who responded to the Lactocare treatment was not significantly more than those who responded to the placebo, there was a two-fold increase in the proportion of those responding to treatment when they had at least 5 years of disease activity compared to the placebo. In a prospective observational cohort study by Caviglia et al. (2021), two strains of *Bifidobacterium* were combined with calcium butyrate and fructooligosaccharides to treat 42 patients with UC in clinical remission over a year. The group receiving the treatment labelled FEEDColon were given the pill twice per day along with their standard of care. A significantly higher proportion of those receiving the combination therapy reached therapeutic success defined as Mayo partial score (MPS) ≤ 2 and fecal calprotectin (FC) < 250 μg/g compared to receiving standard of care alone.

#### 5.3.2 Symptomatic relief and quality of life improvement

The FEEDColon study (Caviglia et al., 2021) showed that those receiving the combination therapy with standard of care treatment experienced significant amelioration of quality of life, abdominal pain, and stool consistency compared to the group receiving standard of care alone. Moreover, following the same study by Kamarli Altun et al., the treatment’s impact on the patients’ quality of life was investigated (Kamarli Altun et al., 2022). Using the short-form 36 (SF-36) questionnaire to assess the quality of life, the treatment group had an insignificant difference in the increase of SF-36 values compared to the control group. However, the treatment group showed a significant increase in the social functioning, mental health, and general health perception scores compared to the control.

The synbiotic studies in IBD patients are presented in Table 3.

**TABLE 3:** Studies evaluating synbioitcs in IBD patients.

Study	Study design	Probiotic intervention	Prebiotic intervention	Subject	Number of patients	Duration of the study	Dosage	Outcome
Kamarlı Altun et al. (2019)	RCT, placebo-controlled, not specified if single or double blinding	*Enterococcus faecium, Lactobacillus plantarum, Streptococcus thermophilus, Bifidobacterium lactis, Lactobacillus acidophilus,* and *Bifidobacterium longum*	Fructooligosaccharides	Mild-to-moderately active UC	40	8 weeks	3 × 10^9^ CFU of the six strains and 225 mg of the prebiotic/tablet; twice per day	-Significant decrease in serum CRP and sedimentation values -Significant improvement in clinical and endoscopic activity. -Significant improvement in clinical parameters compared to the placebo. -Improvements in inflammatory markers and endoscopic activity not significantly different to the placebo group
Amiriani et al. (2020)	RCT, placebo-controlled double-blind trial	*Lactobacillus casei, Lactobacillus acidophilus, Lactobacillus rhamnosus, Lactobacillus bulgaricus, Bifidobacterium breve, Bifidobacterium longum,* and *Streptococcus thermophilus*	Fructooligosaccharides	Mild-to-moderately active UC	60	8 weeks	1 × 10^9^ CFU per tablet of Lactocare, twice per day	-Significant reduction in mean SCCAI clinical activity with Lactocare compared to an insignificant reduction in the placebo group -Insignificant increase in % of those who responded to Lactocare vs. the placebo, but a significant two-fold increase of those who responded when having 5+ years of disease compared to the placebo
Caviglia et al. (2021)	Prospective single-centre observational cohort study	*Bifidobacterium bifidum* and *Bifidobacterium lactis*	Calcium butyrate and fructooligosaccharides	UC in clinical remission	42	1 year	One tablet of FEEDColon twice per day, dosage composition unspecified	-Significantly more reached therapeutic success (MPS ≤ 2 and FC < 250 μg/g) on combination therapy of FEEDColon and 5-ASA standard of care compared to standard of care alone -Significant amelioration of quality of life, abdominal pain, and stool consistency with combination therapy compared to the standard of care group
Kamarli Altun et al. (2022)	RCT, placebo-controlled, single-blind study	*Lactobacillus acidophilus, Lactobacillus plantarum, Enterococcus faecium, Bifidobacterium longum,* *Bifidobacterium lactis,* and *Streptococcus thermophilus*	Fructooligosaccharides	Mild-to-moderately active UC	40	8 weeks	3 × 10^9^ CFU of the six strains and 225 mg of the prebiotic/tablet; twice per day	-Insignificant difference in the increase of SF-36 questionnaire scores compared to the control -Significant increase in social functioning, mental health, and general health perception scores compared to the control

## 6 Conclusion

Based on most of the studies included, there does not appear to be a straightforward answer when it comes to the effectiveness of microbiome modulation in the induction and maintenance of remission of IBD. The selected clinical trials highlight a gap in the IBD research field when it comes to the availability of uniform research protocols with large sample sizes. Most of the studies were limited by having small sample sizes which may not provide the most solid evidence concerning the possibility of commercializing the use of any studied compound. Moreover, most studies did not address the long-term side effects of the combinations used which could provide the basis for future research questions. Another possible limitation is the presence of confounding factors such as variations in dietary habits among IBD patients and differences in medication use which may influence study outcomes.

When it comes to probiotics, the formulations that provided the most favorable results included single strains such as *Lactobacillus rhamnosus* GG and *Bacillus clausii* UBBC-07 as well as multi-strain formulations combining *Lactobacillus* and *Bifidobacterium* species. The aforementioned probiotic formulations were shown to modulate the gut microbiota, reduce pro-inflammatory cytokines, and improve psychological parameters. They also demonstrated clinical and endoscopic benefits. As for the prebiotics, the oligofructose-enriched inulin showed the most promising potential of significantly impacting remission rates with galactooligosaccharides and roasted *P. major* seeds also helping reduce symptomatic manifestations. In terms of the synbiotic studies, most trials included a probiotic core of strains of *Lactobacillus* and *Bifidobacterium* species along with fructooligosaccharides as their corresponding prebiotic with results being fairly promising despite the limited recent studies published.

However, despite these promising effects, the results remained inconclusive due to contradictory findings. While some studies confirmed the ability of probiotics to alleviate clinical symptoms and maintain remission, these benefits were more pronounced in the UC patients. This discrepancy arose due to the unavailability of extensive trials on CD patients, and this population requires further investigation. As for prebiotics, results were also contradictory where some studies showed a significant improvement when using prebiotics compared to controls, yet other studies suggested that while certain improvements were seen, those improvements would also be seen when adhering to standard of care treatment alone. The same applies for the synbiotic studies which pose an even bigger challenge to interpret given the many variables in research protocol, yet the findings are promising and require further investigation as well. It must be noted that while the compounds and organisms mentioned earlier were the most effective, this could also simply be due to most studies happening to cover them over other interventions. The reviews mostly targeted adult patients so the pediatric population could benefit from further investigation. Moreover, more research should be done studying the possibility of utilizing these interventions as a form of primary prevention rather than as an aid to treatment.

A potential area for future research is the personalized formulation of probiotics and prebiotics, or the personalized combination of probiotics and prebiotics to form synbiotics for IBD patients. Such personalized holistic therapy, biotics in combination with other traditional pharmacological therapies, can potentially increase the effectiveness of treatment while also reducing the side effects.

## Abbreviations

IBD, Inflammatory bowel disease; UC, Ulcerative Colitis; CD, Crohn’s Disease; IgA, Immunoglobulin A; SCFA, Short-Chain Fatty Acids; QoL, Quality of Life; BSFS, Bristol Stool Form Scale; SCCAI, Simple Clinical Colitis Activity Index; CDAI, Crohn’s disease activity index; SIBDQ, Short Inflammatory bowel disease questionnaire; IBDQ, Inflammatory bowel disease questionnaire; IBS, Inflammatory Bowel syndrome; HADS, Hospital anxiety and depression scale; LDL, Low-density lipoprotein; FC, Fecal calprotectin; IFN-γ, Interferon gamma; NF-κB, Nuclear factor-kappa B; NO, Nitric Oxide; Il-10, Interleukin 10; TGF-ß, Transforming growth factor-beta; UCEIS, Ulcerative Colitis Endoscopic Index of Severity; AP, Abdominal Pain; SF, Stool frequency; RCT, Randomized controlled trial; CFU, Colony forming units.
